# Commentary: Detecting Cortical Spreading Depolarization with Full Band Scalp Electroencephalography: An Illusion?

**DOI:** 10.3389/fnsys.2018.00019

**Published:** 2018-05-16

**Authors:** Jed A. Hartings, Laura B. Ngwenya, Tomas Watanabe, Brandon Foreman

**Affiliations:** ^1^Department of Neurosurgery, University of Cincinnati, Cincinnati, OH, United States; ^2^Neurotrauma Center at UC Gardner Neuroscience Institute, Cincinnati, OH, United States; ^3^Department of Neurology and Rehabilitation Medicine, University of Cincinnati, OH, United States; ^4^Lannister-Finn Corporation, Bryn Mawr, PA, United States

**Keywords:** spreading depression, spreading depolarizations, traumatic brain injury, electroencephalography, stroke

We read with great interest the study by Hofmeijer et al. (2018) that investigated the potential of scalp EEG to detect spreading depolarizations (SDs) in patients with acute brain injury. Their investigation was prompted by results from the Co-Operative Studies on Brain Injury Depolarizations (COSBID) that described how SDs—identified by strict criteria applied to electrocorticographic (ECoG) recordings from the brain surface (Dreier et al., 2017)—are also manifest in the diverse and complex activity patterns of scalp EEG (Drenckhahn et al., 2012; Hartings et al., 2014). In analyzing over 3,000 h of scalp EEG data, Hofmeijer et al. found no evidence for SD manifestations and thus call into question “the possibility of clinically relevant CSD detection by scalp EEG.” We wish to commend the authors for their contribution and also to raise a few considerations that may bear on the study's interpretation and conclusions.

First, we would like to emphasize that no criteria for definitive diagnosis of SDs by scalp EEG have yet been proposed. The COSBID group has only developed criteria for ECoG recordings from the *brain surface* using subdural electrodes with 2.3-mm diameter (Dreier et al., 2017). These criteria are observation of (1) slow-potential changes, or DC shifts, (2) simultaneous depression of spontaneous activity, if present at baseline, and (3) the spread of these infraslow and higher frequency signatures between electrodes. It is unlikely that these criteria could also be applied in a straightforward manner for SD identification in scalp EEG. Indeed, at our own center, slow potential changes and the spread of depression were found in scalp EEG for only a minority of the SDs that were identified in simultaneous subdural ECoG recordings (Hartings et al., 2014).

Nevertheless, a consistent result obtained in both COSBID studies—conducted in three patient populations (aneurysmal subarachnoid hemorrhage, malignant hemispheric stroke, and traumatic brain injury) in two different countries—was that SDs are manifested in the scalp EEG as depressions of ongoing activity (Drenckhahn et al., 2012; Hartings et al., 2014). These depressions developed and resolved in accordance with the SD patterns confirmed in subdural ECoG recordings: SDs that occurred in relative isolation caused unique EEG depressions followed by recovery, whereas repetitive SDs caused longer-lasting, fused depressions, sometimes lasting hours. In our study, these were the most reliable EEG correlates of SDs, present in 11 of 12 patients and for 81% of all 455 SDs (Hartings et al., 2014). Moreover, the depressions were focal. In view of the simultaneous subdural recordings, we hope that everyone could agree that these findings are not illusory.

Thus, it is interesting that Hofmeijer et al. did not observe similar focal amplitude fluctuations in their cohorts of middle cerebral artery stroke (*n* = 18) and moderate to severe brain trauma (*n* = 18). We expect that the true incidence of SDs would be at least moderate in these groups, based on prior studies in patients with similar injury severities and outcomes (Dohmen et al., 2008; Hartings et al., 2011). Moreover, the EEG techniques used, including Ag/AgCl electrodes, DC-coupled amplifiers, and ≥21-channel montages, were comparable to those used previously.

The authors allude to one important possibility to explain the different findings: all of their patients had intact skulls. By contrast, all patients in the prior studies had undergone neurosurgery to treat their injuries (Drenckhahn et al., 2012; Hartings et al., 2014). The intact skull filters localized (high spatial frequency) brain activity, rendering scalp EEG sensitive only to broadly synchronous rhythms. It is possible after removal of this filter by neurosurgical procedures, and craniectomy in particular, that scalp recordings reflect more focal activity and thus are more sensitive to SD-induced depressions. In addition, it is important to note that SDs have not yet been characterized with verified techniques in a large cohort of non-surgical patients. Although 2 of 5 such patients did have SDs in a recent study (Dreier et al., 2018), SD incidence in these populations is unknown.

Another possibility we would like to suggest is that the method of visual EEG review in 1-h blocks, as used by Hofmeijer et al., may have precluded detection of SD-induced depressions that may have been present. We observed that “isolated” depressions develop quite gradually (interquartile range 8–15 min), are often mild or moderate in maximal extent (IQR 44–67% power reduction), and are prolonged in overall duration (IQR 16–33 min). Such patterns may be particularly challenging to identify in 1-h data segments. Rather, we find that review of data periods up to 8 or even 12 h in highly compressed displays is optimal for visual identification of EEG depression cycles. The longer periods allow a more clear view of deviations from the baseline level, comparison between channels, and identification of recurrent depressions—a pattern that is common for SDs and difficult to attribute to other causes. The Figure 1 illustrates these concepts. In 11 h, there are at least 7 cycles of amplitude modulation in the left hemisphere that are not observed contralaterally. These are difficult to decipher on more expanded time scales (Figures 1B,C).

**Figure 1:**
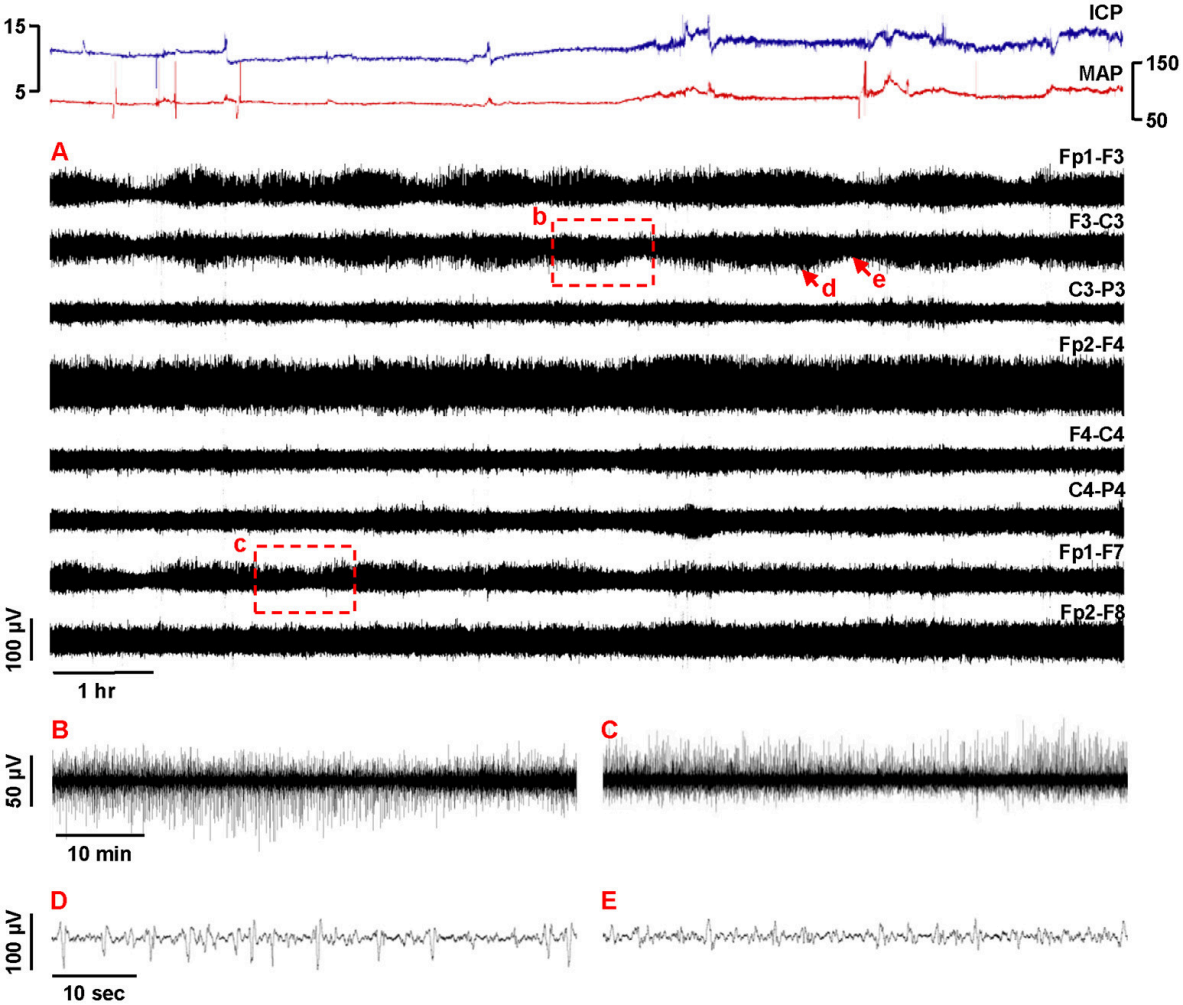
Focal recurrent amplitude depressions in continuous EEG: manifestations of spreading depolarizations? This 56-year-old man fell from a 10-foot ladder. His initial head imaging demonstrated bilateral frontal and temporal contusions, bilateral subdural hemorrhage, and scattered subarachnoid hemorrhage. Post-resuscitation Glasgow Coma Scale score was 10T but he soon decompensated requiring intracranial multimodality monitoring on arrival to the intensive care unit. The following day he underwent bifrontal decompressive craniectomy for refractory intracranial hypertension. His hospital course was complicated by the development of diffuse vasospasm and bifrontal infarcts 2 weeks following his initial trauma, and he was subsequently discharged to hospice care. **(A)** Traces show 11 h of compressed EEG recordings (0.5–50 Hz, bipolar longitudinal montage) following neurosurgery. Breach rhythm was present bilaterally. Several cycles of recurring amplitude depression and recovery are observed in the left hemisphere with prominence at Fp1 and F3. The amplitude depressions are focal, as they are not observed in homologous contralateral recording channels. Furthermore, early cycles (e.g., b and c) are present in both superior (F3-C3) and (Fp1-F7) lateral channels, but later cycles (including those of d and e, and thereafter) are more focally restricted to the superior chain. Amplitude fluctuations are consistent with the magnitude, time course, and repetition of those verified as manifestations of spreading depolarizations, and are not explained by other continuous monitoring variables, such as intracranial pressure (ICP) and mean arterial pressure (MAP). Subdural electrode recordings were not obtained in this patient. With such compressed EEG displays, amplitude changes that are unnoticed or seem insignificant at more expanded time scales can emerge as more distinct, salient, and patterned. **(B,C)** Boxed 1-hr recording segments in **(A)** are shown on more expanded time scales. The amplitude changes readily observed on highly compressed time scales are more difficult to appreciate. **(D,E)** 1-min segments from F3-C3 (arrows in **A**) show the baseline of high-amplitude repetitive discharges and their subsequent suppression as the basis of amplitude fluctuations. Pathologic high amplitude delta activity was present in all patients of our prior trauma series and may be important for SD to be manifested in scalp EEG depressions (Hartings et al., 2014).

Thus, as Hofmeijer et al. acknowledge, it is unknown whether their negative results reflect a limitation of the data analysis methods used, true lack of SD manifestation in EEG of these particular patients, or low or absent SD incidence in the studied populations. These considerations obviate the current lack of criteria to identify SD based on EEG alone and underscore the importance of further studies. At the same time, it seems premature to dismiss the existence of EEG signatures of SD, and we remain optimistic that non-invasive SD detection will be possible. Not the least, we agree with the authors that advanced signal processing could be key to these efforts, and we appreciate their use of Laplacian source derivation which, in principle, may be superior to bipolar montage analysis. It would be useful to know the details of this transformation to facilitate future work and comparison of findings. Again, we thank and applaud the Dutch group for their work and for highlighting important questions and stimulating discussion. We hope that they continue their investigations in this field.

## Author contributions

JH drafted the manuscript and prepared the figure. LN contributed to data collection and study supervision. TW contributed to data preparation and manuscript drafting. BF contributed to data collection and interpretation and figure preparation. All authors edited the manuscript and approved the final version.

### Conflict of interest statement

TW is an employee of the Lannister-Finn Corporation. The other authors declare that the research was conducted in the absence of any commercial or financial relationships that could be construed as a potential conflict of interest.
